# Advances in nanozyme-assisted CRISPR diagnostic technology

**DOI:** 10.3389/fbioe.2026.1796403

**Published:** 2026-02-26

**Authors:** Lang Luo, Yeling Yang, Yubei Zhang, Guobin Mao

**Affiliations:** 1 Department of Biomedical Engineering, Southern University of Science and Technology, Shenzhen, China; 2 Materials Artificial Intelligence Center, Shenzhen Institutes of Advanced Technology, Chinese Academy of Sciences, Shenzhen, China

**Keywords:** biosensing, CRISPR diagnostics, nanozymes, point-of-care testing, signal amplification

## Abstract

The clustered regularly interspaced short palindromic repeats (CRISPR) and CRISPR-associated proteins (Cas) system has significant potential in biological diagnostics because of its precise nucleic acid identification abilities. Traditional CRISPR diagnostics, however, have limitations such as insufficient signal output, dependence on exogenous enzymes, and high equipment demands. Nanozymes, as nanomaterials with enzyme-mimetic catalytic activity, integrate the catalytic efficiency of natural enzymes with the stability and modifiability of nanomaterials, providing a viable resolution to the limitations in CRISPR diagnostics. This article comprehensively evaluates the advancements in nanozyme-enhanced CRISPR diagnostic technologies. Furthermore, it delineates the fundamental attributes of the CRISPR diagnostic system and nanozymes, as well as the necessity of their integration. Moreover, the coupling mechanisms between the CRISPR/Cas system and nanozymes, including the regulation of nanozyme catalytic activity by Cas protein function and CRISPR signal amplification facilitated by nanozymes, were also comprehensively evaluated. The application of this technique in detecting nucleic acid and non-nucleic acid targets was assessed. Further, this study discusses the current limitations of this technology, such as complex separation of heterogeneous systems, laborious reaction protocols, and slow detection rates. The future advancements, such as the establishment of homogenous systems, the creation of integrated devices, and the utilization of single-atom nanozymes, have also been discussed in this review. The results of this study will provide references for the comprehensive integration of nanozymes and CRISPR technology, together with their diagnostic applications.

## 1 Introduction

Biological diagnostic technology is fundamental in areas such as disease prevention and control, food safety monitoring, and environmental quality evaluation, where sensitivity, specificity, and detection efficiency remain focal points of research (Kaminski et al., 2021). Despite their high sensitivity, traditional diagnostic methods like Polymerase Chain Reaction (PCR) depend on complex temperature-controlled instruments, experienced workers, or prolonged amplification durations, complicating the achievement of on-site rapid detection (Yang and Rothman, 2004; Niemz et al., 2011; Chowdhry et al., 2023). In recent years, the CRISPR/Cas system has become a core tool for next-generation diagnostic technology due to its programmable nucleic acid recognition capability and collateral cleavage activity activated by targets, enabling the development of representative diagnostic platforms such as SHERLOCK and DETECTR (Gootenberg et al., 2017; Chen et al., 2018; Chen et al., 2022; Kaminski et al., 2021; Soh et al., 2022; Li H. et al., 2023). However, the CRISPR/Cas system has intrinsic limitations: firstly, the signal output generally relies on the cleavage of fluorescent probes, which necessitates specialized fluorescence detection apparatus, increasing the cost and operational complexity (Kellner et al., 2019; Fozouni et al., 2021; Kaminski et al., 2021). Secondly, certain systems require the addition of natural enzymes in the signal reporting stage, such as horseradish peroxidase (HRP), but these enzymes are susceptible to inactivation and demand stringent storage conditions, elevating detection costs and operational complexity (Gao et al., 2007; Wei and Wang, 2013; Zhang et al., 2021; Babaei et al., 2025).

The identification of nanozymes has introduced a novel method for addressing the aforementioned issues. After the discovery of peroxidase-like activity in Fe3O4 nanoparticles in 2007, nanozymes have been significantly used in biosensing, attributed to their excellent catalytic stability, cost-effectiveness, scalability in synthesis, and easy surface functionalization (Gao et al., 2007; Huang et al., 2019; Jiang et al., 2019; Liang and Yan, 2019; Shamsabadi et al., 2024). Currently synthesized nanozymes include various catalytic forms, such as peroxidases, oxidases, and nucleases, as well as different material systems such as CeO_2_, MnO_2_, MoS_2_, and noble-metal-based nanostructures (Huang et al., 2019; Tian et al., 2025). The integration of nanozymes with the CRISPR/Cas system facilitates the effective amplification of CRISPR recognition signals via the catalytic properties of nanozymes, simplifying the detection process and allowing for visual and cost-effective detection by utilizing the characteristics of nanomaterials (Wu et al., 2022; Arshad et al., 2024). Wu et al. (2022) devised a MnO_2_ nanozyme-mediated CRISPR-Cas12a system that enables colorimetric detection of SARS-CoV-2 by modulating the catalytic activity of MnO_2_ nanozymes via cleavage following Cas12a activation, avoiding the need for a sophisticated detection apparatus (Wu et al., 2022).

In recent years, nanozyme-enhanced CRISPR diagnostic technology has attained significant advancements in target detection range and efficacy (Broto et al., 2022; Shamsabadi et al., 2024). It has evolved from the original detection of nucleic acids to encompass non-nucleic acid targets, including tiny compounds, proteins, and heavy metal ions. The detection sensitivity has been increased to the aM level, and on-site detection modalities, including smartphone help, have been implemented (Chen et al., 2024; Deng et al., 2024; Zhang et al., 2024; Che et al., 2025). This article focuses on “why nanozymes + CRISPR” and systematically investigates the coupling mechanisms, application scenarios, and research advancements of nanozymes and CRISPR, while addressing existing controversies and prospective development trajectories, offering references for further investigation in this domain.

## 2 Coupling mechanism

The effective integration of the CRISPR/Cas system with nanozymes is essential for efficient diagnostics. This process involves modulating the catalytic activity of nanozymes through target recognition by the CRISPR/Cas system, amplifying the recognition signal of the CRISPR/Cas system by leveraging the catalytic properties of nanozymes, and improving detection efficacy through the synergistic assembly of nanozymes and CRISPR.

### 2.1 Regulation of nanozyme catalytic function by Cas protein activity

The primary purpose of the CRISPR/Cas system is to identify target nucleic acids via crRNA and to initiate the nucleic acid cleavage activity of Cas proteins, which includes both the precise cleavage of target nucleic acids and the collateral cleavage activity of non-specific reporter molecules (Kaminski et al., 2021). The Cas12a protein can non-specifically cleave single-stranded DNA (ssDNA) (Chen et al., 2018). Using this attribute, ssDNA can be altered on the surface of nanozymes to function as a “molecular switch.” After target recognition, CRISPR/Cas12a cleaves ssDNA on nanozymes’ surfaces, altering their surface charge, dispersibility, or active-site exposure and thus modulating their catalytic activity (Liu et al., 2015; Zeng et al., 2019). For instance, the MnO_2_ nanozyme-mediated CRISPR/Cas12a colorimetric approach delineated by Wu et al. (2022), wherein the activation of Cas12a triggers the cleavage of ssDNA affixed to the MnO_2_ nanozyme surface, reactivating the nanozyme-catalyzed chromogenic reaction for the detection of SARS-CoV-2 (Wu et al., 2022). Furthermore, Tao et al. (2025) showed a MoS_2_ quantum-dot system in which Cas12a-regulated DNA cleavage on MoS_2_ QDs alters the enzyme-mimicking properties, facilitating sensitive colorimetric detection of viral DNA targets (Tao et al., 2025). Collectively, these examples highlight a common design logic: CRISPR/Cas trans-cleavage acts as the “gate” that rewires nanozyme surface chemistry into a measurable catalytic signal, providing a compact and modular coupling strategy.

### 2.2 Multi-level amplification of CRISPR signals mediated by nanozymes

The improved catalytic activity of nanozymes allows multi-tier amplification of the CRISPR/Cas system recognition signal, addressing the issue of limited signal intensity in the conventional CRISPR/Cas system (Broto et al., 2022; Shamsabadi et al., 2024). This process generally combines CRISPR-mediated target identification with the catalytic activity of nanozymes, creating a sequential “recognition-cleavage-catalytic amplification” response (Babaei et al., 2025). In a standard design framework, crRNA recognition initiates Cas trans-cleavage to cleave various nucleic-acid linkers, releasing numerous nanozymes. Each nanozyme catalyzes multiple substrate molecules to provide enhanced optical or electrochemical outputs (Chen et al., 2023; Xu et al., 2023; Luo et al., 2024).

For example, Zhang et al. (2023) created a nanozyme-mediated amplification-free CRISPR system. It employs the collateral cleavage activity initiated by Cas12a following target recognition to cleave the DNA linker that connects nanozymes to magnetic beads, releasing the nanozymes from the magnetic bead surface. The released nanozymes facilitate the chromogenic process. A single Cas12a can cleave numerous DNA linkers to release multiple nanozymes, with each nanozyme capable of catalyzing several substrate molecules. This results in multi-level signal amplification, enabling the visual detection of target nucleic acids without the need for nucleic acid amplification steps, reducing the detection time to 30 min (Zhang et al., 2023). Mechanistically, this coupling mode emphasizes reporter multiplicity and catalytic turnover rather than solely changing the intrinsic activity of a single nanozyme particle.

### 2.3 Synergistic assembly of nanozymes and CRISPR to improve detection performance

Beyond activity gating and catalytic amplification, because of the assembly characteristics of nanomaterials, the CRISPR/Cas system and nanozymes can be systematically organized into functional complexes, which would increase their synergistic effect and strengthen the specificity and stability of detection (Liu S. et al., 2024; Sui et al., 2025; Zhang X. et al., 2025). In practice, this coupling mode often relies on programmable co-localization (e.g., DNA-guided assembly) or confined microenvironments (e.g., nanostructured scaffolds/nanoflowers) to increase effective local concentrations, suppress nonspecific interactions, and stabilize catalytic activity under challenging conditions.

Jiang et al. (2024) proposed a dCas9-mediated dual-signal platform that facilitates target-dependent assembly of Au–Pt nanozyme units, thus simultaneously amplifying catalytic and SERS responses, which enables high-specificity detection, including single-base discrimination (Jiang et al., 2024). Moreover, Qiu et al. (2024) developed a self-assembled bifunctional nanoflower-based CRISPR/Cas platform for dual-readout detection of *Salmonella enterica*, demonstrating that confined microenvironments and co-encapsulation can increase assay stability and efficacy in complex matrices (Qiu et al., 2024). Overall, this coupling mode highlights that assembly is not merely a packaging step. It can be a functional design lever that improves robustness, reduces interference, and supports integrated readouts, which is particularly relevant for practical deployment.

## 3 Nucleic acid target detection

Nucleic acid testing for pathogenic genomes and clinically significant biomarkers, including both DNA and RNA targets, remains the primary domain of CRISPR diagnostics (Kellner et al., 2019; Broughton et al., 2020). However, molecular diagnostics continues to face limitations, such as low target abundance, matrix-derived interference, and the necessity for rapid, instrument-light readouts appropriate for dispersed testing (Emaus et al., 2020; Budd et al., 2023; Chakraborty, 2024). The incorporation of nanozymes has significantly improved the sensitivity, speed, and on-site application of detection through high-turnover catalytic signal transduction and resilient nano-interfaces (Zandieh and Liu, 2021; Zhao et al., 2023; Wang et al., 2024).

### 3.1 DNA target detection

DNA-target detection includes bacterial genomic markers, viral sequences, and clinically significant biomarkers (Niemz et al., 2011; Kellner et al., 2019; Broughton et al., 2020). Nanozyme-mediated CRISPR assays are particularly beneficial in this field. Collateral-cleavage CRISPR nucleases, such as Cas12a and Cas14a, convert sequence recognition into collateral cleavage, whereas nanozymes transform these molecular events into enhanced colorimetric, electrochemical, or electrochemiluminescent (ECL) outputs (Chen et al., 2018; Harrington et al., 2018; Huang et al., 2019; Li C. et al., 2023; Arshad et al., 2024).

**FIGURE 1:**
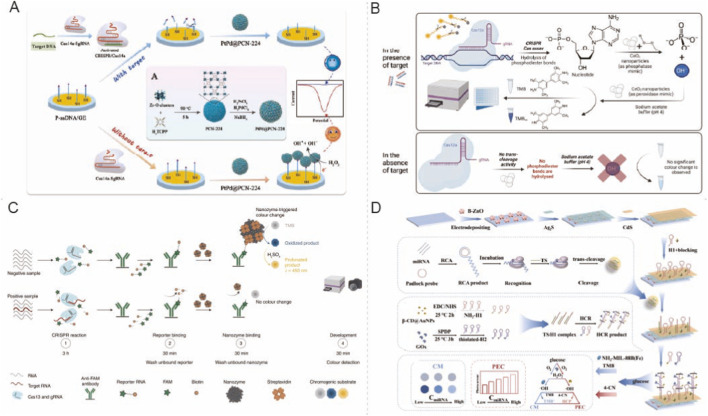
Nanozyme-assisted CRISPR assays for nucleic-acid target detection. **(A)** A signal-off electrochemical CRISPR/Cas14a biosensor in which target-activated trans-cleavage suppresses PtPd@PCN-224 nanozyme assembly on the electrode, resulting in a reduced current response. Reprinted with permission from Li et al. (2023), *Biosens. Bioelectron.* 225, 115098. Copyright (2023), with permission from Elsevier. **(B)** A CeO_2_ nanozyme–mediated RPA–CRISPR Cas12a dual-mode biosensor for *Salmonella invA* DNA, enabling fluorometric and colorimetric outputs. Reprinted with permission from Arshad et al. (2024), *Biosens. Bioelectron.* 247, 115940. Copyright (2024), with permission from Elsevier. **(C)** CrisprZyme, a Cas13-based assay coupled with a Pt@Au nanozyme-linked immunosorbent readout for amplification-free, colorimetric detection of non-coding RNAs. Reproduced with permission from Broto et al. (2022), *Nat. Nanotechnol.* 17, 1120–1126, Springer Nature **(D)** A Cas12a-based multi-amplification microRNA sensing strategy integrating rolling circle amplification and cascade nanozyme catalysis to generate photoelectrochemical and colorimetric signals. Reprinted with permission from Shen et al. (2022), *Sens. Actuators B Chem.* 371, 132585. Copyright (2022), with permission from Elsevier.

Li et al. (2023) presented a CRISPR Cas14a electrochemical biosensor, employing PtPd@PCN-224 nanozymes as catalytic signal tags to enhance the hydrogen peroxide reduction current, demonstrating a characteristic electrochemical format that promotes catalytic signal amplification (Figure 1A). In the presence of target DNA, activated Cas14a initiates trans-cleavage of phosphorylated ssDNA at the electrode interface, inhibiting the assembly of PtPd@PCN-224 on the electrode through Zr-O-P coordination and resulting in a signal-off reduction in current, thus facilitating ultrasensitive detection of pathogenic bacterial DNA (Li C. et al., 2023).

Arshad et al. (2024) proposed an electrochemical transduction approach by creating a CeO_2_ nanozyme-mediated RPA-CRISPR/Cas12a dual-mode biosensor targeting the *invA* gene of *Salmonella* (Figure 1B). This strategy involves the initial amplification of the *invA* sequence via RPA, which then activates the CRISPR/Cas12a-crRNA complex to cleave a FAM-quencher ssDNA reporter, producing a fluorescent signal for sequence-specific identification. The technology employs a CeO_2_ nanozyme-mediated colorimetric readout for orthogonal validation. The technology utilizes the inherent oxidase- and peroxidase-like properties of CeO_2_ in the presence of H_2_O_2_ to produce a target-dependent chromatic transition, providing reliable dual-signal confirmation. This assay attained a minimal detection threshold of 10 CFU/mL and exhibited strong anti-interference characteristics in complex food matrices, including milk and chicken (Arshad et al., 2024).

### 3.2 RNA target detection

RNA-target detection focuses on viral genomes and regulatory non-coding RNAs (Abudayyeh et al., 2016; Barnes et al., 2020; Yang and Patel, 2024). Relative to DNA targets, RNA analytes are generally rarer, more prone to degradation, and frequently considerably smaller, particularly miRNAs (Garneau et al., 2007; Dong et al., 2013; Jet et al., 2021). These attributes require robust signal transduction and efficient background suppression (Dong et al., 2013; Chen F. et al., 2022). For this, nanozymes provide high-turnover catalytic amplification and adaptable readout modalities, enhancing the programmability of CRISPR systems (Thamilselvan and Kim, 2024; Zhang L. et al., 2025).

Wu et al. (2022) presented a MnO_2_ nanozyme-assisted CRISPR/Cas12a platform for the fast detection of the SARS-CoV-2 ORF1ab gene in RNA viruses. The method initially transforms viral RNA templates into double-stranded DNA (dsDNA) intermediates by reverse transcription-recombinase polymerase amplification (RT-RPA). Subsequently, the Cas12a-crRNA complex selectively identifies this DNA, initiating trans-cleavage activity that cleaves ssDNA linkers modified on the surface of magnetic beads, therefore releasing the immobilized MnO_2_ into the supernatant. The released MnO_2_ facilitates TMB color formation through its peroxidase-like activity. This method produces visually discernible outcomes in 45 min, indicating great potential for point-of-care epidemiological surveillance (Wu et al., 2022).

In addition to infections, non-coding RNAs function as essential biomarkers in cancer, although their detection is complicated by their low quantity. Broto et al. (2022) devised the CrisprZyme approach, which combines Cas13/gRNA-mediated RNA detection with an NLISA-type colorimetric output facilitated by extremely efficient Pt@Au nanozymes (Figure 1C). This technique utilizes Cas13 and gRNA recognition to initiate collateral cleavage of a tagged reporter RNA, while the remaining intact reporter binds streptavidin-functionalized Pt@Au nanozymes via biotin contact, catalyzing the oxidation of a chromogenic substrate for quantitative analysis. The authors showed significant applicability across microRNAs, long non-coding RNAs, and circular RNAs in complex samples, including oncological tissue biopsy analysis utilizing circ-AURKA to differentiate between prostate cancer subtypes (Broto et al., 2022).

To improve sensitivity, Shen et al. (2022) integrated miRNA-initiated rolling circle amplification (RCA) with Cas12a trans-cleavage and a hybridization chain reaction circuit, introducing a nanozyme cascade readout that combines beta cyclodextrin-modified gold nanoparticles with an Fe-based MOF nanozyme NH_2_-MIL-88B to promote dual photoelectrochemical and colorimetric outputs (Figure 1D). This multi-amplification system attained sub-femtomolar analytical sensitivity, with detection limits of 0.3 fM for photoelectrochemistry and 0.5 fM for colorimetry, facilitating accurate miRNA differentiation in complex conditions (Shen et al., 2022).

Collectively, the above examples illustrate that nanozyme-assisted CRISPR nucleic-acid assays are evolving from proof-of-concept signal conversion toward designs that explicitly optimize matrix tolerance, detection confirmation, and operational convenience. Therefore, A new platforms can be guided by measurable engineering criteria, including time to result, number of manual handling steps, susceptibility to background in real matrices, and the degree to which the readout is quantitative and portable. This analysis helps clarify why specific architectures are selected for distinct nucleic-acid use cases.

## 4 Non-nucleic acid target detection

Conventional CRISPR/Cas systems primarily focus on nucleic acids. The incorporation of recognition elements such as aptamers has effectively enhanced nanozyme-mediated CRISPR/Cas systems to detect non-nucleic acid targets, including proteins, small compounds, and heavy metal ions, significantly improving the applicability of CRISPR diagnostic technology.

### 4.1 Protein detection

Protein biomarkers are essential for clinical screening and disease management; however, their detection by CRISPR systems is limited by the inability of the Cas-crRNA combination to directly recognize them (Tang et al., 2022; Hartl et al., 2023; Carrasco-Zanini et al., 2024). Therefore, the majority of CRISPR-based protein assays depend on an aptamer or antibody to transform a protein-binding event into the release or production of an activator DNA that can initiate the trans-cleavage of the CRISPR/Cas system (Li et al., 2021; Tang et al., 2022; Paialunga et al., 2025). The nanozyme component functions as the signal output and amplification module, converting CRISPR-mediated nucleic acid cleavage into a visual or electrochemical alteration (Mu et al., 2023).

**FIGURE 2:**
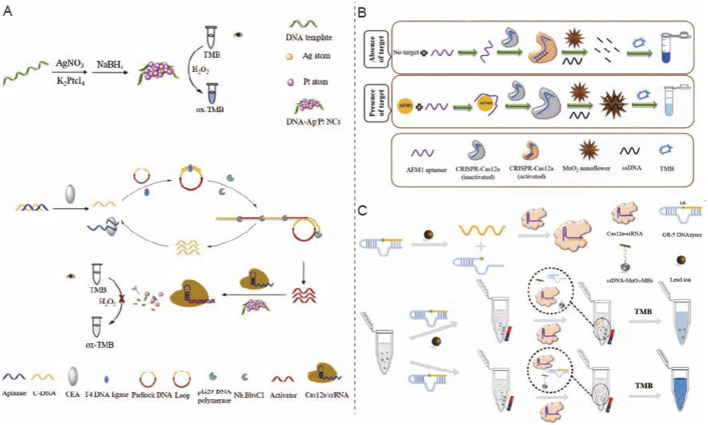
Nanozyme-assisted CRISPR assays for non-nucleic-acid targets. **(A)** A CRISPR/Cas12a-enabled colorimetric biosensor in which rolling circle amplification (RCA) and CRISPR trans-cleavage regulate peroxidase-mimicking DNA–Ag/Pt nanoclusters to generate a TMB-based chromogenic signal. Reprinted with permission from Mu et al. (2023), *Sens. Actuators B Chem.* 375, 132870. Copyright (2023), with permission from Elsevier. **(B)** A Cas12a colorimetric aptasensor for aflatoxin M1 (AFM1) based on ssDNA adsorption-controlled MnO_2_ nanozyme catalysis, translating target binding into an on/off TMB color response. Reprinted with permission from Esmaelpourfarkhani et al. (2024), *Talanta* 271, 125729. Copyright (2024), with permission from Elsevier. **(C)** A Pb^2+^ assay integrating DNAzyme-mediated target transduction with Cas12a activation and MnO_2_ nanozyme reporting, producing a visible TMB colorimetric output. Reprinted with permission from Xu et al. (2023), *Anal. Chim. Acta* 1243, 340827. Copyright (2023), with permission from Elsevier.

Mu et al. (2023) established a visual biosensor for carcinoembryonic antigen (CEA) by integrating rolling circle amplification (RCA), CRISPR/Cas12a, and peroxidase-mimicking DNA–Ag/Pt nanoclusters (NCs) (Figure 2A). The design involves CEA binding to an aptamer complex, which releases a primer strand that promotes padlock probe circularization and initiates RCA, resulting in the production of long repetitive DNA products comprising Cas12a activator sequences; a subsequent nicking step increases the quantity of activator to activate Cas12a. The activated Cas12a subsequently cleaves the DNA scaffold on the Ag/Pt nanoclusters, thus inhibiting the peroxidase-mimetic activity and reducing the UV–vis signal. The assay, employing cascade amplification from RCA and CRISPR, achieved a linear range of 2.5 pg/mL to 2.0 ng/mL, with a detection limit of 0.94 pg/mL, and was verified with serum samples.

### 4.2 Small molecule detection

The identification of small compounds, including mycotoxins, metabolites, and medication residues, is crucial for guaranteeing food safety and enhancing clinical monitoring (Li et al., 2018). Nanozyme-assisted CRISPR platforms address the inherent limitations of small-molecule detection, such as low molecular weight and the absence of multiple binding epitopes, by combining aptamer-based affinity ligands with the trans-cleavage functionality of Cas proteins, converting small-molecule binding events into substantial signals (Niu et al., 2021; Li et al., 2022; Zhu and Zhao, 2023; Liu M. et al., 2024; Zhu et al., 2025).

Wu et al. (2024) integrated a nanozyme–CRISPR cascade into an electrochemical, centrifugal microfluidic system for the detection of ochratoxin A (OTA). Pd@PCN-222 nanozyme catalyzes the reduction of H_2_O_2_, producing a reduction current. In the presence of OTA, competitive binding between OTA and the aptamer strand releases cDNA, which subsequently binds to crRNA and activates Cas12a trans-cleavage; the activated Cas12a then cleaves ssDNA connecting Pd@PCN-222 and the electrode, resulting in a reduction of Pd@PCN-222 at the electrode interface and decreasing the H_2_O_2_ reduction peak current, facilitating preamplification-free detection with a reported detection limit of 1.21 pg mL^−1^. The on-chip incubation and trans-cleavage processes were programmable, allowing on-site workflow integration (Wu et al., 2024).

Esmaelpourfarkhani et al. (2024) established a CRISPR-Cas12a colorimetric aptasensor for the detection of Aflatoxin M1 (AFM1) in a complementary solution-based colorimetric format by manipulating the quasi-oxidase activity of flower-like MnO_2_ nanozymes through a CRISPR/Cas12a system (Figure 2B). In the absence of AFM1, CRISPR/Cas12a is activated and randomly cleaves the ssDNA, restoring the elevated oxidase-like activity of the MnO_2_ nanoflowers and generating a pronounced absorbance signal due to enhanced TMB oxidation. In the presence of AFM1, the CRISPR/Cas12a module remains inactive, resulting in the retention of ssDNA on the nanozyme surface, which inhibits nanozyme activity, restricting TMB oxidation and yielding a low absorbance. The detection limit is as low as 0.05 ng/mL, and the detection outcomes in milk samples correspond with those obtained using high-performance liquid chromatography (Esmaelpourfarkhani et al., 2024).

### 4.3 Heavy metal ion detection

Heavy metal ions (e.g., Pb^2+^ and Hg^2+^) are persistent, non-degradable contaminants that can bioaccumulate and demonstrate significant toxicity at low concentrations, resulting in multi-organ damage. Therefore, there is an urgent need to develop rapid and sensitive detection technologies (Balali-Mood et al., 2021; Pan et al., 2024; Yang et al., 2025). To address the restricted signal transduction and vulnerability to background interference frequently observed in ion-triggered experiments, the integration of the CRISPR/Cas system with a nanozyme readout system improves the amplification of catalytic signals (Xu et al., 2023; Meng et al., 2025).

Xu et al. (2023) developed a DNAzyme-activated, MnO_2_ nanozyme-mediated CRISPR/Cas12a colorimetric protocol for the detection of Pb^2+^ without preamplification (Figure 2C). Pb^2+^ is specifically identified by the GR-5 DNAzyme, which cleaves it at the “rA” site, resulting in the release of a short trigger DNA (tDNA) that subsequently activates the CRISPR/Cas12a system. Activated Cas12a cleaves the ssDNA linker connecting MnO_2_ nanorods to magnetic beads (ssDNA-MnO_2_-MBs), resulting in the cleavage of MnO_2_ and a reduced TMB oxidation signal. The assay demonstrated a linear range of 0.8–2,500 nM, with a stated limit of detection (LOD) of 0.54 nM. Furthermore, specificity can be improved by dual recognition, but sensitivity is increased by MnO_2_ serving as a stable catalytic signal probe that amplifies color output without target preamplification.

Overall, extending nanozyme-assisted CRISPR diagnostic technology to non-nucleic acid targets shifts the central challenge toward reliable molecular transduction. Future platform development is likely to benefit from transducer designs that minimize background, and tolerate complex sample matrices, as well as integrated devices that reduce manual steps while preserving the catalytic gain of nanozyme reporters.

## 5 Conclusion and future

Nanozyme-assisted CRISPR diagnostic technology has transformed biological diagnostics by integrating the complementary advantages of CRISPR/Cas-mediated molecular recognition with nanozyme-facilitated signal amplification, resulting in an integrated “recognition-amplification-readout” framework. This integration provides numerous substantial benefits. First, it facilitates visibility and reduces equipment requirements, as nanozymes transform CRISPR signals into recognizable colorimetric or simple electrochemical outputs, which eliminates the need for complex fluorescence-based equipment. Secondly, it achieves significant sensitivity, with detection limits generally approaching the femtomolar to attomolar range, substantially surpassing traditional CRISPR assays. Moreover, the integration of nanozymes provides increased stability, thus facilitating prolonged storage at ambient temperature. The integration of components such as aptamers extends the technology’s application range, allowing comprehensive detection of both nucleic acid and non-nucleic acid targets.

Despite this potential, the extensive application of nanozyme-assisted CRISPR platforms is restricted by various limitations. A primary issue is the nanozyme’s limited substrate specificity, which can increase background noise and compromise quantitative precision. Moreover, the prevalence of diverse test formats, commonly utilizing magnetic bead-based support, necessitates labor-intensive washing and separation procedures. These restrictions prolong the turnaround time and increase procedure complexity, which are unfavorable to point-of-care testing (POCT). Furthermore, complex biological matrices may undermine analytical accuracy: endogenous redox-active compounds and nonspecific protein adsorption might disrupt the CRISPR/Cas system and nanozyme functionality, resulting in inaccurate responses.

Future advancements in nanozyme-assisted CRISPR diagnostics will largely depend on overcoming various obstacles. At the catalyst level, increasing nanozyme activity and catalytic selectivity via site-isolated or single-atom engineering and customized surface functionalization will be crucial for mitigating nonspecific background interference and enhancing quantitative dependability. Transitioning from bead- or carrier-dependent heterogeneous workflows to homogeneous, separation-free architectures in assay format could significantly improve operations and reduce time-to-result, as demonstrated by target-triggered nanozyme assembly/disassembly or proximity-regulated catalytic switching. Furthermore, matrix robustness is essential for real-sample analysis, as endogenous redox-active species and nonspecific adsorption can disrupt either CRISPR/Cas activity or nanozyme catalysis; therefore, antifouling interfaces, matrix-tolerant buffers, and internal controls must be integrated to mitigate inaccurate responses. The integration of molecular and materials advancements with device engineering and standardized validation will ultimately determine the potential of nanozyme-assisted CRISPR platforms to evolve into widely applicable point-of-care testing solutions in clinical diagnostics, food safety, and environmental monitoring.
